# Economic methodology to preserve the past? Some reflections on *economic theories and their dueling interpretations*

**DOI:** 10.1080/1350178X.2024.2404187

**Published:** 2024-12-20

**Authors:** Catherine Herfeld

**Affiliations:** Institute of Philosophy, Leibniz University Hannover, Hannover, Germany

**Keywords:** Economic modeling, idealization, economic theory, unrealistic assumptions, models as methodological tools, economic appraisal

## Abstract

Methodological appraisal usually aims at a discourse that contributes to the improvement of knowledge production processes in economics. Attempts by economists, such as that by Gilboa et al. (2022. Economic theories and their dueling interpretations. *Journal of Economic Methodology*, 1–20) to engage in such a discourse are laudable and needed because they draw on field-specific expertise and experience from economic practice and thereby can steer such discourse into promising directions. However, while Gilboa et al. seem to share the ambition of doing economic methodology in the service of progress, their contribution is not sufficiently self-critical. Instead of seriously considering the diversity of contemporary modeling practices, their position commits them to an outdated view of economics and can be read as licensing the preservation of conceptual and theoretical elements of standard economics that the discipline has long been criticized for. As such, the authors miss out on fully reaching their own ambitions of a practice-based appraisal that pushes economists towards self-critical and forward-looking modeling.

## Introduction

A methodological paper by a group of first-rate economic theorists reflecting on a core issue in economic methodology is always a welcomed contribution. After all, economic methodologists and philosophers of economics usually have to do quite some arm-twisting to get economists to even listen.^1^ Because one aim of methodological appraisal is to engage and advance debates with a constructive impact on knowledge production in economics, such debates should be informed by questions and considerations of economic practitioners. This is what the authors of the paper *Economic Theories and their Dueling Interpretations* set out to do. Itzhak Gilboa, Andrew Postlewaite, Larry Samuelson, and the late David Schmeidler (2022a) – four well known and highly reputable economic theorists – are explicit about their disappointment that critical discussions about economic models are too far removed from considerations about how those models are used by economists.

To limit the scope of their claims, the authors focus mainly on economic theory as an area they themselves have made major contributions to. Yet, their observations and claims could have more general philosophical implications for debates in the philosophy of economic modeling. They also raise meta-methodological questions about the way philosophers of economics should think about the status of economic models and their relation to economic theory and other economic practices, the approach philosophers of economics should take when appraising those models, and the way in which they should generally be doing philosophy of economics and economic methodology. As such, their paper is a thought-provoking contribution to the methodological debate about the use and usefulness of economic models and the ways in which methodological appraisal and philosophical analysis should be conducted.

The main issue under discussion is one that philosophers and methodologists have been discussing for a while.^2^ It is the puzzling commitment of economists to models that are grounded in highly unrealistic assumptions. Gilboa and colleagues take it that such a commitment frequently appears incomprehensible, even ‘crazy’ (p. 15), especially to non-economists. This is also because the predictive success of those models is moderate at best. In drawing on some of their earlier methodological contributions (Gilboa et al., 2014, 2018, 2022b), the authors want to dissolve this puzzle by clarifying why economists often have good reasons to stick to their models. First, the authors claim that those models can only be appreciated when considering different modeling purposes in economics. They argue that, by taking a closer look into how economists use those models, their critics would see that for some purposes, the assumptions of those models do not necessarily need to be realistic. The authors also point out that, in addition, such models are used for purposes other than the empirical analysis of economic phenomena – such as explanation, prediction, and understanding. Economic models are equally used for theoretical exploration and theory appraisal. This is why economic models should consequently be evaluated with respect to those purposes as well. Second, the authors claim that economists’ reluctance to give up their unrealistic models is because they have a profound appreciation of elegant mathematical modeling results, i.e. results produced with few modeling assumptions (p, 14). This appreciation manifests in a commitment to general theory that goes far beyond the commitment to a model's realisticness, its empirical performance, or its potential to ground interventionist recommendations when informed by that theory.

By making those arguments, Gilboa and colleagues ultimately seem to echo a position already defended by economic theorists, namely that they appreciate economic theory and models grounded in it for their virtues beyond the empirical power of those models. Yet, an innovate aspect of the paper is the attempt to make this position explicit. Aside from providing a general framework for thinking about the relation between standard (micro)economic theory and economic models, the authors also offer an account of models and their purposes that is meant to convince critics that not all economists might be crazy after all. One step they take towards this goal is to illustrate the diversity of economic modeling practices and their purposes by looking more deeply into what economic modelers do, which – once acknowledged – can enable a more focused and nuanced appraisal of economic models.

The meta-methodological position underlying their analysis and account – namely that economic methodology should seriously consider economic practices is, however, one that philosophers and methodologists of economics hardly need to be convinced of. As the field of general philosophy of science, both domains have undergone a practice-turn that is grounded in the view that relevant philosophy of science needs to engage with scientific practices (see, e.g. Hands, 2001). The recent philosophical literature on models and modeling practices stays true to this conviction by reconstructing, distinguishing and carefully analyzing different model types and scientific modeling practices in light of their various purposes (for an overview of the recent practice-based debates in the philosophy of scientific modeling, see, e.g. Knuuttila et al., forthcoming). So, for methodologists and philosophers of economics, claims about the need to consider the diversity of modeling practices to ensure careful and differentiated appraisal do not come as a surprise. To the contrary, the goal of making this diversity explicit is compatible with a large set of recent contributions to the literature on models and modeling in philosophy of science generally and in philosophy of economics particularly.

In this spirit, I do not question the authors’ goal to offer the basis for a practice-based and thus more effective methodological appraisal of economic models that takes the diversity of modeling practices as its premise. To the contrary. I wholeheartedly agree with their attempt to put this goal highest on the methodologist’s agenda. For a long time, before the practice turn, critical discussions and methodological appraisal often remained too abstract to inform economic practice, too far away from the problems the practitioners are concerned with, and too much at risk of provoking or even fueling misunderstandings and confusions about the subject at hand. For which purposes practitioners apply their models is an essential aspect of evaluating those models and consequently should always be the starting point for any constructive critique and for providing relevant and forward-looking methodological guidance to economics. The authors are at great pains to support their position by engaging with a variety of concrete examples of economic models beyond the usual suspects, such as Schelling’s model of racial segregation or Akerlof’s model of the markets for lemons, the two standard go-to cases in the philosophical literature. By illustrating the existing diversity of modeling purposes and discussing its importance for methodological critique in the debate about unrealistic assumptions, the authors emphasise the important point that attacking economic models simply for being based on ‘unrealistic’ assumptions independent of their purpose is not constructive.

I take the attempt to call out those accounts that rest upon a simplified view of economic models and those that undertake unconstructive methodological appraisal to already be an achievement. However, in this comment, I want to point out two serious weaknesses in the authors' own methodological account that potentially undermine their goal. First, I argue that their portrayal of contemporary economics as being rooted in an all-encompassing commitment to economic theory is at best a partial, if not outdated view of economics and of economic modeling practices today. Specifically, because their rather one-sided characterization of economics as being still mainly theory-centered, their account ultimately fails on a more substantive level to capture the diversity of current economic modeling practices. Consequently, they thereby also seem to neglect that the critique of unrealistic assumptions might have become more relevant for some of the kinds of modeling practices in economics today that the authors do not consider. Second, the account is at risk for being used to license an uncritical preservation of the general framework of standard economics, ultimately shielding utility theory and equilibrium analysis from empirical scrutiny. More specifically, instead of using models for fostering empirical and methodological analysis that advances progressive recommendations for the field, the authors' account may serve to justify a status quo that economists have long been criticized for maintaining. Both issues weaken the authors' goal to illustrate the importance for methodological appraisal to seriously engage with contemporary economic modeling practices, while also being constructive – for an appraisal that could genuinely push economics forward as a science.

### A sketch of the framework

Before getting to both points, let us first look more closely at the authors’ account of economic models. At the heart of their position that using models grounded in unrealistic assumptions can be legitimate in economics is a set of distinctions that together serve as a framework for the authors to make the diversity of models, of modeling practices and of modeling purposes in economics explicit. The main distinction they introduce is between three types of models, namely between positive models, normative models and analytical models. These model types differ primarily in how the models belonging to those categories are used. Positive models are used for empirical purposes, normative models are used for prescriptive purposes, and analytical models are used for the methodological purpose of analyzing economic theory. Models in all categories are grounded upon economic theory.

As well will see, the category of analytical models is the most important one in order to follow the authors’ explanation for economists' acceptance of unrealistic assumptions. Analytical models have the methodological purposes of appraising economic theory (p. 5). They are used to analyze the structure of, and explore the implications of modeling results for, economic theory (p. 5). By providing a way of studying mathematical relations within a model that is itself primarily informed by economic theory, analytical models are methodological tools by either allowing for proof-of-concept analyses or for conceptual and theoretical exploration. The authors contrast analytical with positive models, which are used for explanations or predictions, and with normative models, which are used for the purpose of formulating (policy) recommendations or prescribing interventions.

Gilboa and colleagues seem to suggest that every economic model can generally be placed in one of the three model type categories. To which category any one model belongs is decided by the economist as she chooses her intended modeling purpose. A model does not come with an inherent interpretation but rather a model's interpretation depends upon its purpose. Thus, the models in the distinct categories differ with respect to their purpose and, respectively, with respect to their interpretation. Moreover, the interpretation of one and the same model can change when its purpose changes. As such, the same model can have more than one of the three interpretations and, in principle, may belong to any of these categories at different times or in different contexts of use.

Deciding on a model's purpose and, in consequence, its interpretation is ultimately up to the individual scientist. As such, ‘the preferred interpretation [of one and the same model] might vary from one economist to another, and even the same researcher might prefer different interpretations in different contexts or at different times' (p. 7). But the decision of a model's purpose depends, in turn, on the context of application, ‘the surrounding economic literature, and the argument made by the interpreter’ (p. 4 and 11). As such, in which category a model belongs is decided upon individually, is highly contextual, and can change over time. That a model can be given different interpretations makes models highly flexible. Categorical criticism of models for being unrealistic without differentiating between the distinct modeling purposes and, respectively, between the different interpretations they get as a consequence, is not constructive on this view. As a demand for more realistic assumptions can be meaningfully formulated only towards positive but not necessarily analytical or prescriptive models, a constructive criticism of unrealistic economic models has to be preceded by a careful analysis of the respective model type to be appraised.

Underlying this three-fold distinction between model types lies a philosophically rather underspecified understanding of scientific theories, of the nature of models, and of the relation between the two. Gilboa and colleagues differentiate between a framework, a theory, a set of model and their results, which they take to be hierarchically ordered from general (the framework) to concrete (the results). On their view, all of economics starts with a general ‘conceptual framework’ that grounds every economic ‘theory,’ ‘model’ and ultimately ‘result.’ Results are (formally) derived from more general models, which are themselves special cases of even more general scientific theories. Economic theories are in turn part of one and the same conceptual framework. Defined as ‘a formal structure that doesn’t specify the real-life objects to which its entities relate,’ (p. 16, en. 6) a conceptual framework is in a first instance detached from a particular interpretation. A conceptual framework is a purely formal entity that allows for various interpretations of its concepts and, in combination with a set of instructions for how to apply the framework, yields multiple theories that ground models, which can have ‘dueling interpretations’. According to the authors, such a framework can but does not have to be identified with one single theory, which is possible because economic theories on their view are mathematical (or even axiomatic) theories that are in the first instance uninterpreted.

On the authors’ account, the general conceptual framework is that of utility theory, i.e. that agents maximize an objective function subject to constraints, and of equilibrium analysis, i.e. the (comparative) analysis of equilibria that result from the interaction of maximizing agents (p. 1). All economic theories and thus models are conceptually grounded in this framework according to Gilboa and colleagues. For example, expected utility maximization is part of this conceptual framework. It provides a general ‘language and tools for analysis’ and only becomes a refutable theory when its conceptual elements, such as states, outcomes, acts and the utility function itself are further specified (p. 16, en. 7). Importantly, because those specifications can have distinct forms, the framework can result in very different theories. In this specification process from framework to theories, utility maximization interpreted as, for example, maximizing expected material payoff is then used as a theory to ground the construction of different kinds of models. Those expected utility models then can have a descriptive, analytical or a normative interpretation, depending on the way they are used.

As a concrete example to illustrate how theories, models and results relate to the general conceptual framework, the authors discuss Paul Milgrom and Nancy Stokey’s so-called no-trade theorem (Milgrom & Stokey, 1982). The no-trade theorem is a theoretical model, which yields the result that under certain conditions, information asymmetries alone are not sufficient to establish trade; they can give rise to a situation where no trade takes place. This theorem is formally derived from a model of a competitive exchange economy that is grounded upon the (unrealistic) behavioral assumption that agents maximize expected utility under given constraints. As such, the model rests upon a specified variant of the theory of utility maximization in which utility maximization is specified in terms of the maximization of material benefit, given market prices. The general use of utility theory, in turn, is an application of the more general conceptual framework of agents being conceptualized as utility maximizers; the concept of utility and the object of maximization remain unspecified at this general conceptual level.

That theoretical models in economics – such as the Milgom and Stokey model – have been criticized before has partly been due to a long-standing commitment among economists to the view that models are primarily predictive tools. According to this view, the realisticness of modeling assumptions is largely irrelevant for making good predictions. However, the authors want to oppose such a one-sided perspective. This is why they complement their distinction between the three model types by a set of additional distinctions that are meant to illustrate the diversity of models and model usage in economics. For instance, Gilboa and colleagues distinguish between using positive models for the purpose of prediction *and* for the purpose of explanation. The authors thereby stress a point – namely, that explanation is equally important as a modeling purpose for economists as prediction that has long been underhighlighted, possibly also due to the influence of Milton Friedman’s (1953) famous essay defending prediction as the main modeling purpose in economics. Indeed, especially in more applied fields, economists frequently sacrifice predictive power for providing explanations and generating an increased understanding of the economic world through the identification of causal relationships (e.g. Chetty, 2015, Rodrik, 2015).

The authors introduce another relevant distinction – between case-based and rule-based reasoning – to further illustrate why false modeling assumptions might not matter for predictive purposes.^3^ According to Gilboa et al., economic models are often used for case-based as opposed to rule-based reasoning. In rule-based reasoning, a reasoner formulates general rules or theories about identified generalizations to make predictions about the world (Gilboa et al., 2014). On this account, models provide statements about regularities and allow for prediction via deductive inference. Depending on whether the prediction is supported by the data, this way of reasoning would have consequences for economic theory. In contrast, in case-based (or analogical) reasoning, a reasoner does not aim at finding general regularities to make predictions but rather produces predictions via analogies. To address a prediction problem, the economist identifies past cases that have successfully dealt with similar problems to make predictions about the present case (Gilboa et al., 2014). On this view, a model functions as a theoretical case, which enables predictions via analogies alongside other cases (p. 9). Cases can be of different kinds: real, experimental, and theoretical; as such, they do not have to, but they can be theoretical models alongside, and complemented by, other cases such as experiments, computer simulations, etc.^4^ Usually, economists favor theoretical cases, i.e. models that are simple. However, theoretical cases come without specifications of the similarity relation to other cases or further instructions of how to use models in case-based reasoning. This is why economists have to make this similarity judgement themselves.

More specifically, when models are only informed by theory, they might turn out to be highly idealized. Yet, their role is no less important than that of cases, which are informed by empirical data or set up as experiments. They can reveal what could possibly occur, given certain conditions. However, the usefulness of such a theoretical model for making predictions about real situations – prediction as being mainly defined by the accuracy of fitting yet-unobserved data (p. 7) – depends upon the scientist’s judgement about how similar the idealized agents in the model are to the real agents in the situation and about how similar the model situation of the market is to a real market. A theoretical case is usually not a real situation but comparable to a thought experiment about an economic problem the economist makes a case-based prediction about. Because such an approach amounts in a first step to a theoretical analysis, this way of using models as analogies – especially also when grounded in unrealistic assumptions – does not aim at refuting economic theory and should thus not be criticized for failing to do so, according to the authors.

As indicated, the framework that Gilboa and colleagues sketch raises a host of general methodologically relevant issues and questions and most of them cannot be addressed in this comment. However, before elaborating on the two aforementioned points in greater detail, I will make one more general comment, namely that in the same way it is desirable that philosophers and methodologists engage with economic practices, it is desirable that methodological considerations should substantially engage with the philosophical literature. While the authors offer their general account as a methodological contribution and thereby correctly highlight the variety of modeling purposes in economics, it is somewhat disappointing that their account is philosophically rather impoverished. Their elaboration about model-based explanations is a case in point. It is the latest when the authors identify 'scientific explanation' with experiencing an ‘a-ha- moment' that produces a ‘warm feeling inside’ (p. 7) that a philosophically informed audience will find their account wanting.

Philosophers have made extensive efforts to explicate what scientific explanation (including the problem of explaining with highly idealized theoretical models) amounts to in economics (e.g. see Verreault-Julien, 2021 for a survey, part. Sect. 2). The discussion around the epistemic benefits of so-called ‘minimal’ theoretical models, i.e. models that lack any similarity or resemblance relation to the world (Batterman & Rice, 2014), and particularly about their explanatory power and epistemic benefits (e.g. Grüne-Yanoff, 2009, 2013, Fumagalli, 2015, 2016), is thus only one example for a philosophical debate that the authors could have more seriously engaged with to showcase how philosophy of economics could benefit a methodological discussion among economists by carefully working out in greater detail when the realisticness of modeling assumptions is necessary.^5^

In the remaining sections, I focus on the account of economic models and their relation to theory to address the questions whether the authors’ observations still apply to contemporary economics and in which way this account, particularly the use of analytical models as methodological tools, enables progressive methodological appraisal. First, I propose that Gilboa et al. do not do justice to all major modeling practices in economics today. By focusing exclusively on theory-based modeling specifically understood, they ignore the substantial changes that modeling practices have undergone in the aftermath of the applied turn. Second, I claim that their framework together with their model typology provides the ground for licensing a preservation strategy of standard economic theory and thereby seem to justify precisely the kinds of immunization strategies that economists have long been criticized for.

### Accepting an outdated view of economic modeling practices

The first issue refers to the theory-centric view of economics that Gilboa and colleagues take as their departure point. Apart from the fact that their view of economic theorists as users of models that apply them regularly for methodological purposes might be overestimating the methodological ambitions of economic theorists, the authors’ claims describing the current state of economics as one in which economic models are derived from standard economic theory seems to rest upon an at least partially outdated portrayal of economics. As such, this view raises questions about the relevance of their considerations for understanding modeling practices in economics today. One reason for characterizing economics as a strongly theory-centered field might be that the authors are themselves economic theorists, possibly engaging mainly with the economic theory community. However, as I am going to elaborate below, the authors thereby seem to ignore that the role of economic theory has decreased and that economic modeling has become a sometimes even separate endeavor.

To be fair, I should start by stressing that Gilboa and colleagues are, of course, not the last pure theory warriors of the economics profession. They rightly emphasize that economists have for a long time found value in theories and specific mathematical results (p. 2). Certainly, the epistemic and even aesthetic values economists hold on to – such as unification, simplicity, elegance, and internal consistency – doubtlessly still influence theory and modeling choices in economics today. In more theoretical branches, economists remain open or even favor sacrificing other epistemic values such as accuracy and predictive success for a unifying theory that is consistent with the general conceptual framework of equilibrium analysis and utility theory, which has been in place the latest since the nineteenth century. In this sense, the authors’ position also resonates with long existing philosophical contributions showing that economists unificationist ambitions could explain their strong commitment to economic theory and their expansive tendencies behind the phenomenon of ‘economics imperialism’ (e.g. Mäki, 2001, 2009). However, the authors’ view that economic theory takes on the role of grounding most of today’s economic modeling practices seems to be an overstatement.

First, an exaggerated emphasis on the importance of economic theory manifests in the way in which the authors generally characterize the relationship between scientific theories and models. While theoretical models can certainly stand in a logical relationship with economic theory and, indirectly, with the more general conceptual framework of utility maximization and equilibrium analysis, a core function of economic models has been to mediate between theory and the world (Morgan & Morrison, 1999). On this pragmatic view, models are not mere applications of theory but are instead autonomous entities with their own structure, purpose, and function. Models constitute sources of knowledge in providing understanding in ways that theories alone cannot. More specifically, models can occupy a variety of functions: While one of their functions is to enable the development, exploration, and theories, the application of a theory within a model is pragmatic—it adapts to the needs of the particular context, which may involve empirical data, computational considerations, or the intended purpose of the model. In this capacity, some economic models interact with, but are independent of, and thus are not merely deducible from, economic theory. One could argue that this pragmatic account of models as autonomous tools is just one among many but not necessarily the most convincing one to characterize economics. And, of course, the philosophical literature on models and their relation to theory is, extensive and different accounts of this relationship on offer are still under dispute among philosophers (for a discussion of the semantic, syntactic and pragmatic accounts, see Morgan & Morrison, 1999, ch. 1–3). Yet, given the importance that philosophers of science place on the role of scientific practices today, an account of models according to which they are always logically related to scientific theory is increasingly seen as being one-sided or even outdated. This has been convincingly illustrated to be particularly characteristic of economics (e.g. Morgan, 2012; Morgan & Morrison, 1999).

Second, recent discussions among economists and historians of economics support a more complex view of the relationship between a theoretical framework and models than the one Gilboa and colleagues suggest (see, e.g. Morgan, 2012; Backhouse & Cherrier, 2017). First, while economic theory – including utility theory – still plays a central role in economics today, empirical analysis have supported the hypothesis that the days of the discipline's exclusive focus on economic theory are over (Hamermesh, 2013). There is also a more extensive engagement by economists in a wide array of empirical practices (Hamermesh, 2013; Rodrik 2015). Economists are increasingly engaged in applied research and empirical analyses, including econometric studies, various field and laboratory experimentation techniques, and randomized controlled trials. In many cases, economic theory is entirely absent from these activities (Biddle & Hamermesh, 2017). Similarly, that theory increasingly takes a backseat in contemporary economics holds for many modeling practices pursued in economics today. Theoretical models are complemented and partly even replaced by a large set of other kinds of modeling techniques that often do not have any relation to theory at all.

This decline of economic theory has been discussed under the label of the ‘applied turn’ in history of economics (Backhouse & Cherrier, 2017). It has been argued that in the past 30 years or so, publishing pure theory papers became increasingly difficult in top economics journals. Furthermore, to capture the changing role of theory, it has been argued that the relationship between theoretical and empirical research has undergone a profound redefinition (Backhouse & Cherrier, 2017, p. 5). This definitional change is also reflected in the much tighter connection between the formulation of a theoretical model and specific empirical problems, which has been analyzed under the label of ‘applied theory' (see Backhouse and Cherrier (2017) for a discussion of the term and the historical shift towards ‘applied theory’).

It is not only that the omnipresence of economic theory and its role to ground economic models has diminished. The applied turn also manifests in the decreasing prevalence of analytical models in economics of the sort that Gilboa and colleagues have in mind. Economists surely still use theory to inform their models and they also use analytical models to appraise economic theory. In those cases, the critique of unrealistic assumptions might indeed be beside the point. However, analytical models are less widespread in economics than 30 or 40 years ago. Instead, a lot of what is economic modeling nowadays can be said to fall into the category of positive models. Because those models are used to explain and predict phenomena of interest for economists, the fact that they are grounded in unrealistic assumptions might be a legitimate issue, depending on the target system they are applied to.

This should be an issue of concern for the authors because the scope of target systems that economists apply their models to has been drastically extended during the second half of the twentieth century.^6^ One reason for why many economists discussed but did not prioritize the attempt to ground, for example, behavioral assumptions in economic theory upon more sophisticated psychological concepts and results already long before the 1970s and 1980s was that they, like the authors, did not take more realistic assumptions to be necessary to theorize about the phenomena economists were concerned with. Marginalist thought and twentieth century neoclassical economics – the intellectual roots of the conceptual framework that Gilboa and colleagues take to be grounding all of economics today – had a strong focus on studying aggregate phenomena located at the level of the market. In the 1960s, economists like Kenneth Arrow or Gary Becker showed that for deriving the theorems representing the most important economic relationships such as the law of supply and demand, more accurate behavioral assumptions were not necessary (e.g. Arrow, 1958; Becker, 1962). Only when – from the 1980s onwards – an increasing interest in individual decision-making arguably led to a broader set of *explananda* related to human choice, economists began to seriously consider rethinking economic theory in light of psychological findings. More specifically, when economic models – often based upon rationality assumptions – were taken to be positive models and applied to this extended set of problems about choice, they were frequently considered to be too unrealistic, which led in turn to the renewal of the critique of unrealistic assumptions mainly voiced by behavioral economists.

Today, explaining and predicting (economic) decision-making lies at the heart of what economists are interested in. This is why an appraisal of positive models might require a more serious engagement with the critique of unrealistic assumptions, depending on the purpose of the model. Downplaying the relevance of critique today might result from a neglect of not only the increased use of positive models that are not derived from theory but also of the fact that those positive models are applied to explain and predict human behavior in all its facets, which makes a large set of those models in contemporary economics legitimately the target of this critique. While this observation does not undermine the general point of the authors, this neglect furthermore questions the relevance of their account for thinking about economic modeling techniques and for coping with the critique of unrealistic assumptions in economic models today.

In sum, the account of economic models presented by Gilboa and colleagues is intended to set the stage for highlighting the diversity of the economist’s modeling toolbox. By distinguishing between positive, normative, and analytical models, it already becomes clear that economists are engaged in a variety of empirical, theoretical, conceptual and also policy-related issues. However, by focusing exclusively on theory-based modeling that is always grounded in the same conceptual framework, their appraisal seems to, on the one hand, ignore and on the other hand reduce the de facto existing diversity of modeling practices in economics today to a view of the framework-theory-model relation that was dominant in the past. Their account neglects that theory and modeling practices have changed in the aftermath of the applied turn in economics, implying that they downplay the potential consequences of the critique of unrealistic assumptions for a lot of modeling practices in economics today. Given that the scope of target systems has been extended in economics and that today, individual choice is a major explanandum in economics, the criticism of unrealistic assumptions might apply more frequently to more positive models, mainly those that rest upon unrealistic behavioral assumptions. In those cases, the critique should be taken seriously.

All this is not to say that economic models that are based upon theory nowadays are not still grounded upon the same conceptual framework of utility maximization and equilibrium. While some philosophers of economics have claimed that ‘we're all behavioral economists now' (Angner, 2019), I agree with the authors that economic models, even those models we would *prima facie* categorize as conceptually distinct from neoclassical models, such as behavioral models usually still rest upon the standard theoretical framework that Gilboa and colleagues sketch.; in that sense, it is questionable whether behavioral economics rests upon more realistic assumptions. The authors' claim is that ‘theoretical behavioral economics, for the most part, retains the conceptual framework of maximization-and-equilibrium and [only] differs from standard theories either by using non-material determinants of utility or by allowing errors and biases in reasoning’ (p. 2). While I agree, I would argue that any proper methodological appraisal of such positive models would have to be grounded in a case-by-case assessment to see on whether the critique applies, depending on the target system that the model is applied to.

### Licensing theoretical conservatism

It should be acknowledged that Gilboa and colleagues' account of economic models is a first step towards setting the stage for highlighting the diversity of modelling techniques in the economist’s toolbox. Those efforts to further explicate what model diversity in economics amounts to should be appreciated. However, their commitment to a rather narrow focus on theory-based economic modeling might ultimately be counterproductive towards the authors’ self-set goal of seriously grounding methodological appraisal of economic models in actual modeling practices. This is because their account – particularly their distinction between empirical and analytical models – could be used for licensing a continuous and unlimited preservation of the general conceptual framework of utility theory and equilibrium against empirical scrutiny. Such a move would stand in stark contrast with the general aim of economic methodology of ensuring appraisal that is (ultimately) done in the service of making progress in economics, i.e. that contributes to the continuous improvement in knowledge production processes in economics. This is the second issue I want to elaborate.

The authors emphasize the importance of analytical models and their methodological function in economics. It even seems that, for them, the usefulness and appreciation of analytical models is what explains economists’ long-lasting commitment to models with unrealistic assumptions. Gilboa and colleagues do not offer a clear definition of analytical models. They illustrate what they mean by this model type with an example of a physical model, namely a maquette of a town square. Such a maquette can describe an existing square or one that existed in the past (as positive models do); it can describe a proposed design for a non-existing square (as normative models do); and they can test the feasibility of a possible square. This last role of checking whether something that is represented by the model is possible is usually that of analytical models. More specifically, analytical models have the main function of enabling proofs of concept. They enable logical consistency checks, i.e. allow for checking whether there is one model in a class of models whose assumptions can exhibit some stylized fact about some phenomena that could actually or possibly occur. Analytical models equally allow economists to check whether some stylized fact can be derived from those assumptions that captures a phenomenon whose occurrence in the real world is entirely implausible. Because those models are firmly grounded in utility theory and the equilibrium framework, they can be used to prove ‘that there exists a formal mathematical structure within which all agents are rational, have commonly known beliefs, and exhibit a certain behavior pattern [which] is an exercise that proves the consistency of the assumptions with some stylized facts’ (p. 5). It is at least in this sense that analytical models can be used to critically examine economic reasoning and, more importantly, economic theory.

To operate in such a functional capacity, analytical models require a commitment to the general conceptual framework of economic theory and its standard assumptions. The examples provided by the authors to illustrate this are plenty, one of them being again the no-trade theorem. The model provides a consistency check by showing that a statement about a pattern in the world – namely that no trade will be initiated among traders in the market – necessarily follows from a set of highly unrealistic assumptions about traders. Yet, the model proves a result that is implausible as a characterization of actual markets (where trade is always initiated). In consequence, the model should – according to the authors – not be understood as making a claim about trading in actual (financial) markets. Rather, because in this case an anomaly follows from the standard economic modeling assumptions and ultimately from the conceptual framework underlying it, the found consistency with an anomalous result should be understood as questioning standard economic theory. It is in this way that an analytical model serves as methodological tool to appraise economic theory.

The authors’ view that analytical models serve as methodological tools could – principally – be one way of regularly putting economic theory under critical scrutiny. More specifically, their’ distinction between models used for prediction versus models used for critique further addresses how we can understand that analytical models can fall into the category of methodology. Gilboa and colleagues differentiate economics (dealing with economic phenomena) from economic methods (referring to instruments that economists use to study economic phenomena) and from economic methodologies (tools which are used to study scientific phenomena of economics). This three-fold distinction is useful because it helps us to see that tools belonging at least to the last two categories serve different purposes and ignoring this differentiation can lead to confusions about model purposes being either empirical or methodological. Naturally, on the authors’ account, which models serve as economic methods (empirical and normative models), and which serve as tools from economic methodology (analytical models), crucially depends on a model’s interpretation. Moreover, one and the same model can either be used as methodological tool or as method to study some real-world phenomenon. As aforementioned, a model’s interpretation is individually chosen, depending on contextual features of the model’s purpose, among other things. This choice is partly grounded in intuitions and plausibility considerations of the individual scientist; there is no thus principled way by which the two – models as methods and models as methodological tools – can be distinguished. Because scientists can change a model’s interpretations as they switch its purpose, the function of a model is partly contingent upon the interpretation it gets assigned at some point in time by a scientist.

On the authors’ account, an economic model that is interpreted as an analytical model could play a substantial role in theory appraisal. Gilboa and colleagues point out that ‘illustrating the consistency of a theory with a given phenomenon’ by using an analytical model is a practice that is more common in economics than elsewhere (p. 6). If the modeling results are used for theory appraisal, this practice could indeed be effective in economic theory development. Ideally, an analytical interpretation of an economic model is chosen in order to undertake such consistency tests. One result of this test is a methodological claim to the effect that the underlying conceptual framework and theory are supported or not, depending on whether the consistency of the standard assumptions with the stylized fact is established and whether the actual occurrence of the phenomenon represented is plausible or even actually observable. An inconsistency result or one where the assumptions are consistent with a highly implausible stylized fact like in the case of the Milgrom-Stokey theorem, should then have immediate consequences for the underlying theory and more generally the conceptual framework. If an analytical model proves a highly implausible result, we expect the model’s main methodological contribution to be to substantiate critical conclusions about the empirical usefulness of economic theory. The consequence would be to revise or even replace the underlying theory and the general conceptual framework to build better models.

However, Gilboa and colleagues’ account contains two elements of contingency that can lead to serious challenges to proceeding in this way. The first element of contingency enters because the authors' account allows model interpretations to change depending on context. Economists could switch between different interpretations as they please in the moment where a model is not fit for an initially intended purpose. Because the status of a model as either positive, normative, or analytical depends on the goal of, and the ascribed interpretation by the modeler, it is in their hands to make this decision. That the category into which a model falls is decided by the scientist when it is used is possible because a model on the authors' account has in a first instance no interpretation attached to it. The example of the maquette illustrates what that means: ‘There is nothing in the maquette itself that can unambiguously determine which the intended interpretation is (among the above and possibly others). The same maquette can be constructed to illustrate the square to people who cannot visit it, to propose a reconstruction of a square that looks quite different at present, or to test whether suggested buildings can fit into the space’ (p. 4).

If the model turns out not to be suitable for one of the three purposes, we can thus easily switch the model’s interpretation. For example, if predictions of an empirical model cannot be supported by the data, we can turn the model into a normative one by giving it a different interpretation and present its purpose as being prescriptive. On this account, empirical models become normative or analytical just when a scientist wants them to. Such flexibility allows that a modeler can backtrack from a model's original purpose but preserves the model by re-interpreting it despite its failure to fulfill that purpose. A famous example is Leonard Savage’s overnight switch from an empirical interpretation of subjective expected utility theory to a normative interpretation when realizing his own (and other people's) failure to act according to his rationality axioms (p. 6; see also, e.g. Guala, 2000; Moscati, 2016). The second element of contingency arises only in the case of analytical models. Whether a stylized fact derived in the model can be viewed as an economic phenomenon that can plausibly occur in the actual world crucially depends on the current state of the world and, more importantly, on the scientist's assessment of how similar the stylized fact and the actual world are.

Both elements of continency open the door for two kinds of dangers. First, empirical models can any time be turned into normative or analytical models because their predictions are not supported by the data. This flexibility might be utilized as a strategy to protect the theoretical core of standard economics. As the authors themselves point out, if a descriptively interpreted model and its assumptions are inconsistent with the data, the economist – particularly when ‘facing an aggressive audience’ (p. 7) – can always proclaim that ‘rather than promoting the model as an explanation of a real-life phenomenon,’ it was used as a proof of concept or an exercise in testing the ‘scope of the standard paradigm,’ with open consequences for the theory (p. 7). According to the authors, turning empirical into analytical models is much more common in economics than turning them into normative models because ‘the switch from a descriptive interpretation of a model .. to a consistency test .. is more continuous and less dramatic than between descriptive and normative interpretations’ (p. 6). However, while such a switch might be what usually happens in economics, the possibility of following this strategy would also reduce the importance of carefully working out well-functioning empirical models that can be used for explanation and prediction. Rather, the option of turning empirical into normative and particularly analytical models by switching their interpretation could lead to protecting the theoretical core of standard economics against empirical scrutiny.

Second, because an answer to the question whether analytical models show an inconsistency depends on the researcher’s judgement about whether some result is plausible or not, economists might either not judge the result as describing an anomaly or avoid the last step, namely to undertake substantial theoretical modifications or even replace a theory in light of such a result. The exercise of providing a consistency check is most effective, of course, if the idea is to show that some possibly arising anomaly is either consistent with the standard modeling framework, or leads to the revision or replacement of the framework. Yet, in reality, particularly those observations that appear inconsistent with a given theory do only sometimes and, if at all, only very slowly lead to theoretical modifications in economics. Even Gilboa and colleagues do not seem to consider the move towards substantial theory adjustment or replacement as being a necessary consequence. Although effective appraisal is not in principle precluded on their account, the view that analytical models are used for methodological appraisal does not ensure that they are used in the service of making progress. Rather, the account opens the door for providing justifications of a continuous use of standard economic theory by allowing for the kind of lukewarm and ad hoc theory adjustment practices that economists have engaged in, and have been criticized for throughout a long period of time.

Both kinds of dangers are profoundly problematic. Being able to make an individual decision to turn a given model that, for example, does not live up to standards of validity or shows that predictions cannot be empirically supported, into an analytical model opens the door for keeping empirically obscure modeling practices. Furthermore, if an analytical model is used for consistency checks that, in case of an anomalous result, almost never has an effect on theory construction and change, using them for methodological purposes seems to be a methodological strategy that is prone to misuse. Both dangers reveal that the account by Gilboa and colleagues can easily be instrumentalized to preserve the profession’s unconditional commitment to the standard conceptual framework of economics. This can be done by, first, turning unsuccessful empirical models into normative or analytical models and by, second, either neglecting anomalies that result from consistency tests by not judging the stylized facts derived to be implausible or by just ignoring the demand to revise standard economic theory in light of such a resulting anomaly. Yet, it is precisely this persistent and almost dogmatic preservation of the general conceptual framework of utility theory and equilibrium in light of the fact that economic models often rest upon unrealistic assumptions that critics of economics have been pointing out for a long time as preventing economics from progressing.

Both dangers reveal some of the reasons why the account proposed by the authors implies a methodological position that is too risky, too narrow, and a too minimalist to defend: risky, because it could be used to legitimize an old-fashioned practice of preserving standard economic theory and its conceptual framework rather than using models to challenge and revise existing theory and conceptual framework alike when appropriate; narrow, because if methodological appraisal primarily consists of checking for consistency of modeling assumptions with a certain actual or possibly stylized fact, and if consistency only amounts to answering the question whether the assumptions can actually produce a pattern in the model that is plausible, then a lot of what we take to be an essential function of economic methodology – and of methodological principles beyond that of consistency – would not be considered. While it is not clear whether Gilboa and colleagues want to go that far, their position does not automatically appear to present a view that is more forward-looking and inclusive. Rather, it could be used to license theoretical conservatism in economics.

Finally, the account is philosophically minimalist in that Gilboa and colleagues operate with a rather one-sided view of scientific models, which could have substantially benefitted from an actual engagement with the philosophical and historical literature on economic modeling. Two examples should suffice to see this. First, philosophers of economics have made serious and careful attempts to show how we can learn from highly idealized theoretical models that are grounded in unrealistic assumptions. In those discussions, philosophically informed arguments in favor of the position implicitly underlying the authors’ account of analytical models are given. Grüne-Yanoff (2013), for example, has argued that, albeit being inadequate representations of real-world target systems, we can learn from models that are grounded upon unrealistic assumptions because they tell us something about the possibility or impossibility of certain patterns or mechanisms produced by the model actually occurring in our world. They thereby impact our confidence in certain hypotheses we were committed to about what could possibly or would actually occur. While we find references also in the authors’ earlier methodological research to this philosophical literature, it is disappointing that there is no serious engagement with such existing philosophical proposals that have clear overlaps with the authors’ positions.

Second, the debate about scientific models based on unrealistic assumptions has been frequently cast in philosophy of science as a debate about different kinds of idealizations, the degree to which models are idealized and the utility of such idealized models. Weisberg (2013), for example, has made an extensive effort to argue that the practice of idealization in science, i.e. the practice of intentionally distorting models that are meant to represent some target system, is relative to their intended targets. What gets idealized in a model depends on the goal of the modeler and how the model is used. This shows how the authors tackle an important issue and push for a debate that is not unique to economics but is relevant for thinking about the use and usefulness of highly idealized models in science generally. It might be argued that this debate does not concern analytical models directly because, according to Gilboa and colleagues, the purpose of analytical models is ultimately not to represent some target system. Yet, it does concern the authors’ category of positive models. Idealizations are chosen in order to isolate relevant causal relationships that can support model-based explanations. Idealizations have to be carefully justified and, at least in some cases, they come with the explicit goal to de-idealize models in the long run. However, for Gilboa and colleagues, the matter of idealization seems to be different. Choosing idealizations is not so much determined by the representational capacities of the resulting model but rather by the general conceptual framework that is given. Instead of justifying the assumptions through their capacity to isolate causal relations, they serve as unjustified departure point pre-set by economic theory and the underlying conceptual framework. This practice of choosing model idealizations does not stand in the service of ensuring the epistemic utility of positive models but rather of keeping the general conceptual framework intact.

Against this background, the account defended by Gilboa and colleagues allows for analytical modeling practices grounded upon unrealistic assumptions to be useful as methodological tools. However, the authors do not necessarily ensure progressive theoretical adjustments or the formulation of calls for theory replacement in light of empirical anomalies to make theory more empirically powerful. We are left with the question how economic methodology could proceed in a way that puts analytical models into the service of a more progressive theory development and adjustment. Moreover, while it is true that analytical modeling, qua its function, is not necessarily threated by the critique of unrealistic assumptions, for the more expansive practice of using positive models to the purpose of explaining and predicting choice, the critique – as extensively discussed by behavioral economics – can have more serious consequences for models and economic theory alike.

Let me sum up. What the authors seem to implicitly take as a framework for their observations about economics is philosophically rather critical. Although they do not fully embrace it, they seem to take Lakatos' theory of research programs to give a possible account of what might regularly happen in economics; scientific theories are not refuted immediately considering empirical counterevidence. Rather, the underlying conceptual framework is used to develop theories further: ‘the central paradigm [is] to be defended by a protective belt' (p. 1). This is a view of theory change in economics that is old-fashioned and can easily lead to dogmatism. Making the point of preserving the conceptual framework as the central ‘paradigm’ of economics without considering the extensive secondary literature in philosophy of science discussing the problems of applying Lakatos to economics (see, e.g. Hands, 2001, for a discussion), their considerations read not only like an outdated way to describe economic practices today but also like a justification of the past, not pushing sufficiently that a science also advances by seriously and continuously question its theoretical basis.

Of course, descriptive claims about the profession require evidential support. While the authors are explicit about primarily embracing a descriptive, ‘sociology of science viewpoint' to characterize the current state of modeling practices in economics, Gilboa and colleagues make clear that at this point they ground their empirical claims only in daily observations, personal experiences and anecdotal evidence from regular interactions with their peers (p. 2). This lack of evidence is unfortunate because the plausibility of the authors’ general account and framework strongly hinges upon the characteristics of economics that the authors identify. It also raises doubts about whether their attempt to spark a more constructive methodological discussion can be successful. Rather, they seem to thereby miss out on addressing modelers beyond the community of economic theorists. As a such, a thorough sociological analysis of data on the economics profession, including their current modeling practices and the ways they justify their methodological commitments, represents just another one of the additional research avenues their analysis implicitly points us to.

## Conclusion

The contribution by Gilboa and colleagues is a call for carefully considering actual economic modeling practices in critical discussions, methodological appraisal and philosophical critique. Their point that we can see why economists hold on to a – sometimes even considered to be ‘crazy’ – practice of using models grounded in highly unrealistic assumptions if we consider the diversity of model types and modeling practices is well taken. Furthermore, because interpretations of a model might shift when used for different purposes, it is important that we consider the context of application and the common experience of the modeler in our assessments of the model’s plausibility and its justification. To ensure a forward-looking methodological appraisal of economic models that allows for taking progressive steps in economics today, however, an even broader view of the role of economic theory, of existing types of economic models and of kinds of modeling practices would be required. Furthermore, in order to set an example of a practice-based methodology and philosophy of economics, formulating a clearly articulated methodological rule for reinterpreting economic models, would have strengthened Gilboa and colleagues' call. Such an approach would demonstrate how a practice-based methodology and philosophy of economics might address a critical issue in economic modeling while simultaneously contributing to progress in the discipline.

## Notes

1 Some examples of notable exceptions are Deaton and Cartwright (2018) and Rodrik (2015).

2 Different aspects of the debate have been discussed over the last decades by, e.g., Green and Shapiro (1994), Hausman (2023) Lehtinen and Kuorikoski (2007), Rosenberg (1992). For a survey of the different kinds of critiques particularly focused on unrealistic behavioral assumptions, see Herfeld (2022).

3 A more elaborate version of this distinction is introduced in earlier work by the authors (Gilboa et al., 2018).

4 The authors define ‘theoretical’ work in economics as relying ‘primarily on logical or mathematical arguments, and as ‘empirical’ if it works primarily with data, including administrative data, experiment results, historical documents, survey responses, and so on’ (Gilboa et al., 2022b, p. 898 f.).

5 In fairness, the authors cite a few protagonists in debates around those issues particularly in previous papers (Gilboa et al., 2014) but do not extensively engage with this literature to work out their own account in contrast to existing ones.

6 Partly, economics and philosophers of economics have already captured this extended scope of problems as a trend towards ‘economics imperialism’ (Mäki, 2009).
